# Mutation assay using single-molecule real-time (SMRT^TM^) sequencing technology

**DOI:** 10.1186/s41021-015-0017-5

**Published:** 2015-09-01

**Authors:** Tomonari Matsuda, Shun Matsuda, Masami Yamada

**Affiliations:** Research Center for Environmental Quality Management, Kyoto University, Shiga, Japan; National institute of Health Sciences, Tokyo, Japan; Tomonari Matsuda, Research Center for Environmental Quality Management, Kyoto University, 1-2 Yumihama, Otsu, Shiga 520-0811 Japan

**Keywords:** PacBio RSII DNA sequencer, Single-molecule real-time (SMRT) sequencing technology, Mutation assay

## Abstract

**Introduction:**

We present here a simple, phenotype-independent mutation assay using a PacBio RSII DNA sequencer employing single-molecule real-time (SMRT) sequencing technology. *Salmonella typhimurium* YG7108 was treated with the alkylating agent *N*-ethyl-*N*-nitrosourea (ENU) and grown though several generations to fix the induced mutations, the DNA was extracted and the mutations were analyzed by using the SMRT DNA sequencer.

**Results:**

The ENU-induced base-substitution frequency was 15.4 per Megabase pair, which is highly consistent with our previous results based on colony isolation and next-generation sequencing. The induced mutation spectrum (95% G:C → A:T, 5% A:T → G:C) is also consistent with the known ENU signature. The base-substitution frequency of the control was calculated to be less than 0.12 per Megabase pair. A current limitation of the approach is the high frequency of artifactual insertion and deletion mutations it detects.

**Conclusions:**

Ultra-low frequency base-substitution mutations can be detected directly by using the SMRT DNA sequencer, and this technology provides a phenotype-independent mutation assay.

## Introduction

**Fig. 1:**
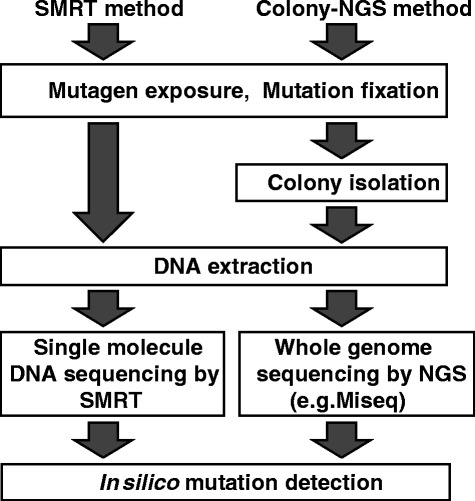
Two distinct strategies to detect low-frequency mutations using high-throughput DNA sequencers

Mutation assays capable of detecting somatic mutations at very low frequencies are important in the areas of environmental mutagenesis, carcinogenesis, epidemiology, and regulatory science. They are especially important in the context of safety evaluation of newly developed drugs or industrial chemicals. Although many mutation assays have been developed, most rely on some kind of phenotypic selection, which involves time-consuming procedures and is potentially biased. We previously reported a phenotype-free mutation assay using next-generation DNA sequencing [1]. In that study, we treated a *Salmonella typhimurium* strain with a mutagen to induced and fix mutations, followed by colony isolation and whole-genome sequencing of the colonies. The induced mutations were successfully detected *in silico* using bioinformatics software. That strategy is summarized in Fig. 1 and named the ‘Colony-NGS method’. Although the approach is simple and reliable, difficulties still remain when it is applied to mammalian cells. This is because: 1) the colony-isolation step is much more technically challenging in the case of mammalian cells compared to bacterial cells, and 2) the mammalian genome is diploid and hundreds of times larger than the bacterial genome, which limits deep coverage in sequencing. Furthermore, the Colony-NGS method is not applicable to bio-monitoring of somatic mutations in tissues of experimental animals or clinical specimens from patients because it is impossible to do the colony isolation from those sources.

Recently, ‘Duplex Sequencing’ methodologies, which enable detecting a single mutation among >1 × 10^7^ nucleotides by using a general next-generation DNA sequencing (NGS) technology, have been developed [2,3]. This is a very promising strategy for application to bio-monitoring of somatic mutations. However, here in this paper we demonstrate an alternative approach by using single-molecule real-time sequencing.

The PacBio RS II DNA sequencer (Pacific Biosciences, Inc.) is a recent innovation [4] based on a single-molecule real-time (SMRT) technology. Since it is able to read the sequence of a single DNA molecule, it can in principle detect the mutations present in the molecule just by sequencing it accurately, as summarized in Fig. 1 (named the ‘SMRT method’) [5]. A significant advantage of this strategy is that the colony isolation step is unnecessary, so that the approach should be applicable to any cell line and specimen from experimental animals, patients and environmental animals.

However, a drawback of this technology is the accuracy of the sequencing data it generates. At present, the error rate in raw reads of the PacBio sequencer is exceedingly high (~15%). To help overcome this problem, the ‘SMRTbell^TM^ template’, in which single-stranded DNA loops are ligated to both ends of a double-stranded DNA, is used to direct sequencing of the same DNA molecule repeatedly [6]. The greater the number of repeat reads so as to generate a consensus read of multiple sub-reads from the same single circular DNA template – i.e., a circular consensus sequence (CCS) read – the more accurate the sequencing result [7]. In this study, we validated that we can detect ultra-low frequency mutations by using the SMRT method with the CCS strategy.

## Materials and methods

### Materials

ENU (CAS No. 759-73-9) and dimethyl sulfoxide (DMSO; CAS NO. 67-68-5) were purchased from Wako (Osaka, Japan). The test strain *Salmonella typhimurium* YG7108, *hisG46 rfa* Δ*uvrB bio ada*_ST_::*kan*^r^*ogt*_ST_::*cat*^r^, which is highly sensitive to alkylating agents, was used in this study [8].

### Mutagen exposure and mutation fixation

The exposure method followed the Ames test 20-min pre-incubation procedure [9]. The YG7108 strain was cultured overnight at 37 °C in nutrient broth (No.2, OXOID) containing 25 μg/mL kanamycin and 10 μg/mL chloramphenicol. Phosphate buffer (0.5 mL), DMSO or 2.5 mg/mL ENU (0.1 mL) and the overnight culture (0.1 mL) were mixed in a tube in that order and incubated for 20 min at 37 °C with gentle shaking at 100 rpm. A 1-μL portion was added into 10 mL of LB medium and cultured at 37 °C for 13 h to fix mutations, after which DNA was extracted. The rest of the mixture was poured onto a minimum agar plate in 2 mL of 0.6 % soft agar and incubated for two days at 37 °C, following which the revertant colonies were counted.

### Preparation of SMRTbell^TM^ templates and sequencing

The genomic DNA samples (5 μg each) were sheared to 50-1000 bp (average 280 bp) fragments by using a Covaris Shearing Device, and used to construct a PacBio DNA library using a SMRTbell Template Prep Kit 1.0 following the manufacturer’s guidelines (http://www.pacb.com/samplenet/PC_250bp_Amplicon_Library_Preparation_and_Sequencing.pdf). Each sample was sequenced on the PacBio RS platform on a single SMRT Cell with C2-P4 chemistry. The base calling and CCS read generation was carried out using PacBio’s instrument control and SMRT Analysis software.

### In silico mutation detection

Mutation detection was carried out by using CLC Genomics Workbench software (ver 7). The fastq files of raw data and CCS were imported into the software. The CCS fastq files were mapped to reference *Salmonella* genome sequences: NC_003197 (*S. typhimurium* str. LT2 chromosome, complete genome, 4,857,432 bp), and CP003387 (*S. typhimurium* str. 798 plasmid p798_93, complete sequence, 93,877 bp). The point mutations were detected using the Basic Variant Detection command (first screening). The essential parameters of the Basic Variant Detection were: ploidy = 1, minimum coverage = 1, minimum count = 1, minimum frequency (%) = 0.1, neighborhood radius = 5, minimum central quality = 40, minimum neighborhood quality = 40. The mutated reads were searched in the CCS fastq files and their corresponding raw reads were extracted from the raw-fastq files. The extracted raw reads were combined in a new fastq file and mapped to the *Salmonella* reference sequence again. The raw reads were manually checked and mutation calls were counted with the help of the viewer function of the CLC Genomics Workbench software.

## Results

**Fig. 2:**
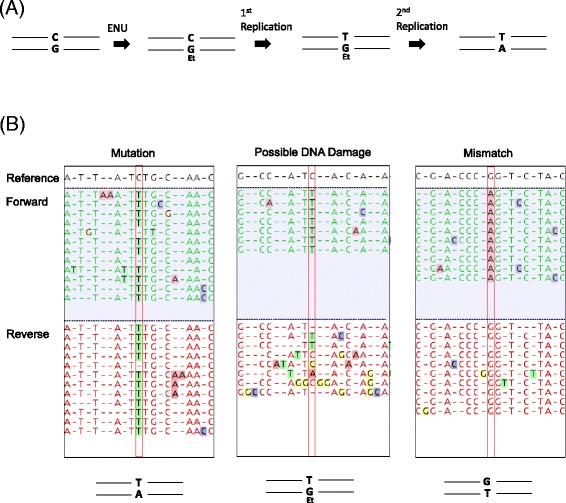
Detection of mutations, DNA damage and mismatches by mapping of raw reads of the SMRT sequencer. **a** Example of ENU induction of an alkylated base (O^6^-ethyl-guanine) in genomic DNA, which will induce a G to A mutation after the 2^nd^ round of replication. **b** Examples of mapped reads. In cases of a real mutation, the same base is clearly called in both the forward and reverse reads. In cases of DNA damage, one strand is mapped clearly but the other strand is not. In cases of mismatch, both the forward and reverse reads are mapped clearly but different bases are called between the forward and reverse reads

The test strain *Salmonella typhimurium* YG7108, which is highly sensitive to alkylating agents, was treated with ENU (Fig. 2a) or its solvent DMSO, followed by dilution and growth overnight in LB medium to fix mutations. Genomic DNA was extracted from the overnight culture. SMRTbell templates were prepared from the DNA samples, with an average insertion size of 280 bp. Note that no PCR amplification step was carried out during preparation of the SMRTbell templates, which is essential to minimize the occurrence of artifactual mutations. The templates were subjected to the sequencing reaction in the PacBio RS II platform, and fastq files were generated from the raw data (contains all the sequence information of multiple sub-reads) and CCS data (contains only the consensus sequence). The threshold of the CCS was a pass time (the number of times the same molecule was repeatedly read) of 10 and 99% accuracy.

The CCS-fastq files were imported to CLC Genomics Workbench software (ver.7). In total, 8.09 and 8.56 Mbp of the sequence data were obtained from the control and ENU-treated samples, respectively. The CCS reads were mapped to the reference sequence of *Salmonella typhimurium* and the point mutations were detected *in silico*. Improbably large numbers of insertions and deletions were called in both the control (405 insertions and 424 deletions) and ENU-treated (367 insertions and 1276 deletions) samples, respectively (Table 1). We had previously analyzed mutations induced in the same bacterial strain with the same exposure protocol by isolating colonies and carrying out whole-genome sequencing. In that previous study, we analyzed the entire genome of each of 4 clones (4.8 Mbp of *Salmonella* genome × 4 clones = 19.6 Mbp search region), but did not detect any insertions and deletions in either the control or ENU-treated samples (unpublished observations). Thus we concluded that the insertions and deletions called in this present study are not reliable and most probably artifacts. In the case of base substitutions, however, 19 and 160 mutations were called in the control and ENU-treated samples, respectively (Table 1). While these frequencies are consistent with the results of our previous study, they are still higher than the estimated values. Thus we decided to proceed with a confirmation step regarding the base substitutions.

**Table 1:** Number of mutation-calls at the first screening

Sample	No. of bases analyzed (Mb)	No. of mutations called		
		Insertions	Deletions	Base substitutions
Control	8.09	405	424	19
ENU	8.56	376	1276	160

Next, we obtained sequence IDs of the CCS reads in which the base substitutions were called at the first screening. Then we searched the sequence IDs in the raw fastq files and extracted the corresponding information of the sequence IDs, and made new fastq files which contained the raw repeated sequence data of the molecules in which the base substitution was possibly present. The newly edited fastq files were mapped to the same *Salmonella* reference sequence. Typical examples of mapped raw reads are shown in Fig. 2b. In the sequencing reaction using the SMRTbell template, the plus and minus strands of a double-stranded DNA molecule are read alternately, thus almost equivalent numbers of forward and reverse reads were obtained. In cases of real mutations, the same base substitutions will be called in both the forward and reverse reads. In cases where different base substitutions were called between the forward and reverse reads, these must be templates bearing a mismatch. In cases where a specific base was clearly called for on one strand but a variety of bases was called for the opposite strand, this may indicate the existence of persistent DNA damage.

After carefully checking the raw data, the base substitution mutations called in Table 1 were counted again and shown in Tables 2, 3 and 4. After recalculation, the numbers of ‘real’ base substitution mutations were 0 and 132 in the control and ENU-treated samples, respectively (Table 4). The rest were likely due to mismatches, DNA damage, SNPs that the strain originally possessed, calls at the edges of the mapped read which did not have sufficient coverage, and so on.

**Table 2:** Details of the 19 base substitutions called at the first screening in the control sample

Reference position	Reference	Forward read				Reverse read				Comment	Judgement	*p*-value^**^
		Most dominant allele	Coverage	Read count	*p*-value^*^	Most dominant allele	Coverage	Read count	*p*-value^*^			
999271	C	C	33	23	3.9E-20	T	36	30	5.5E-31		Mismatch	0
4778252	T	T	17	16	9.1E-19	A	19	19	4.4E-23		Mismatch	0
3355477	0047	C	13	12	4.4E-14	G	11	10	1.0E-11		Mismatch	1.0E-11
536849	C	C	9	9	2.5E-11	T	9	9	2.5E-11		Mismatch	5.0E-11
1051080	G	A	10	9	1.6E-10	G	10	9	1.6E-10		Mismatch	3.1E-10
3287776	A	T	11	8	8.2E-08	A	12	11	6.7E-13		Mismatch	8.2E-08
3823422	C	C	7	4	5.7E-03	G	11	10	1.0E-11		Mismatch	5.7E-03
316363	G									edge of map	No	
694963	C									edge of map	No	
918766	G									edge of map	No	
1922859	C									edge of map	No	
4423790	C									edge of map	No	
4144134	C									No mutation	No	
4515279	C									No mutation	No	
290717	C									original allele	No	
1760048	A									original allele	No	
1760052	A									original allele	No	
3741045	T									original allele	No	
4099877	G									original allele	No	

^*^Probability that the real allele is not the most dominant allele

^**^Probability that the Judgement is not correct

**Table 3:** Details of the 160 base substitutions called at the first screening in the ENU-treated sample

Reference position	Reference position	Forward read				Reverse read				Comment	Judgement	*p*-value^**^
		Most dominant allele	Coverage	Read count	*p*-value^*^	Most dominant allele	Coverage	Read count	*p*-value^*^			
146824	G	A	22	19	1.2E-20	A	22	18	9.9E-19		Mutation	0
994061	G	A	20	19	2.9E-22	A	20	17	2.4E-18		Mutation	0
2007634	G	A	33	24	5.9E-22	A	31	25	2.9E-25		Mutation	0
2044677	C	T	24	19	3.9E-19	T	21	18	1.7E-19		Mutation	0
2724713	C	T	32	21	3.4E-17	T	33	22	2.5E-18		Mutation	0
2747120	G	A	34	24	2.9E-21	A	37	31	4.0E-32		Mutation	0
2871399	G	A	32	26	2.1E-26	A	34	28	1.1E-28		Mutation	0
2930794	G	A	26	23	2.8E-25	A	24	18	3.0E-17		Mutation	0
3007696	A	G	45	33	4.8E-30	G	47	36	4.0E-34		Mutation	0
3322100	C	T	21	18	1.7E-19	T	22	19	1.2E-20		Mutation	0
3666060	G	A	29	23	5.7E-23	A	30	22	2.2E-20		Mutation	0
3695370	G	A	20	17	2.4E-18	A	21	18	1.7E-19		Mutation	0
3708252	A	G	29	26	9.5E-29	G	31	25	2.9E-25		Mutation	0
3863986	G	A	18	16	5.8E-18	A	19	16	3.5E-17		Mutation	0
3961843	G	A	25	20	2.8E-20	A	25	23	4.3E-26		Mutation	0
4320817	C	T	21	18	1.7E-19	T	21	18	1.7E-19		Mutation	0
2171812	G	A	16	15	1.3E-17	A	17	15	8.4E-17		Mutation	1.1E-16
327560	C	T	23	17	4.3E-16	T	24	20	4.9E-21		Mutation	4.4E-16
2209612	A	G	16	14	1.2E-15	G	15	14	2.0E-16		Mutation	1.4E-15
2705366	G	A	24	17	2.2E-15	A	24	22	6.3E-25		Mutation	2.2E-15
2215678	C	T	30	19	6.1E-15	T	30	26	6.0E-28		Mutation	6.1E-15
3881583	C	T	15	13	1.8E-14	T	14	14	3.1E-17		Mutation	1.8E-14
1368298	G	A	16	13	1.1E-13	A	17	17	9.5E-21		Mutation	1.1E-13
4840145	G	A	16	13	1.1E-13	A	18	14	4.2E-14		Mutation	1.5E-13
390064	C	T	17	13	6.1E-13	T	19	16	3.5E-17		Mutation	6.1E-13
733247	C	T	17	13	6.1E-13	T	18	16	5.8E-18		Mutation	6.1E-13
3257503	G	A	17	13	6.1E-13	A	17	16	9.1E-19		Mutation	6.1E-13
935658	G	A	18	15	5.0E-16	A	17	13	6.1E-13		Mutation	6.1E-13
2316694	C	T	17	14	7.4E-15	T	17	13	6.1E-13		Mutation	6.2E-13
414142	G	A	12	11	6.7E-13	A	12	12	6.9E-15		Mutation	6.7E-13
556175	G	A	13	12	4.4E-14	A	12	11	6.7E-13		Mutation	7.1E-13
355651	C	T	38	30	1.8E-29	T	36	20	7.3E-13		Mutation	7.3E-13
748721	C	T	14	12	2.7E-13	T	12	11	6.7E-13		Mutation	9.4E-13
2715604	C	T	20	14	1.2E-12	T	24	20	4.9E-21		Mutation	1.2E-12
2504585	C	T	10	10	1.6E-12	T	10	10	1.6E-12		Mutation	3.2E-12
688445	G	A	12	11	6.7E-13	A	11	10	1.0E-11		Mutation	1.1E-11
222807	C	T	18	16	5.8E-18	T	19	13	1.7E-11		Mutation	1.7E-11
4652102	G	A	19	13	1.7E-11	A	22	20	1.3E-22		Mutation	1.7E-11
3117258	G	A	25	19	2.2E-18	A	22	14	3.0E-11		Mutation	3.0E-11
1005055	C	T	16	12	8.9E-12	T	14	11	2.4E-11		Mutation	3.2E-11
2264426	G	A	11	10	1.0E-11	A	9	9	2.5E-11		Mutation	3.5E-11
992465	C	T	14	11	2.4E-11	T	14	11	2.4E-11		Mutation	4.7E-11
1076365	G	T	12	10	6.1E-11	T	12	11	6.7E-13		Mutation	6.2E-11
458994	C	A	29	26	9.5E-29	A	23	14	1.4E-10		Mutation	1.4E-10
3062433	C	T	18	14	4.2E-14	T	13	10	3.5E-10		Mutation	3.5E-10
421079	G	T	8	8	4.0E-10	T	9	9	2.5E-11		Mutation	4.2E-10
4736812	C	A	24	14	6.4E-10	A	18	16	5.8E-18		Mutation	6.4E-10
2957288	C	T	12	12	6.9E-15	T	11	9	9.4E-10		Mutation	9.4E-10
3872165	C	T	11	9	9.4E-10	T	10	10	1.6E-12		Mutation	9.4E-10
278409	G	T	19	12	1.2E-09	T	23	14	1.4E-10		Mutation	1.4E-09
2861538	G	A	15	13	1.8E-14	A	14	10	1.9E-09		Mutation	1.9E-09
1408682	G	A	9	8	2.4E-09	A	9	9	2.5E-11		Mutation	2.5E-09l
272653	C	A	10	9	1.6E-10	A	9	8	2.4E-09		Mutation	2.6E-09
2757635	G	T	9	8	2.4E-09	T	8	8	4.0E-10		Mutation	2.8E-09
4148066	C	A	9	8	2.4E-09	A	8	8	4.0E-10		Mutation	2.8E-09
206275		T	17	11	3.6E-09	T	14	11	2.4E-11		Mutation	3.6E-09
250264	C	T	9	8	2.4E-09	T	9	8	2.4E-09		Mutation	4.9E-09
2425294	C	T	9	8	2.4E-09	T	9	8	2.4E-09		Mutation	4.9E-09
4431921	G	A	12	9	5.4E-09	A	12	10	6.1E-11		Mutation	5.4E-09
909863	C	T	31	16	5.5E-09	T	31	17	9.6E-11		Mutation	5.6E-09
1085221	G	A	12	9	5.4E-09	A	12	9	5.4E-09		Mutation	1.1E-08
2250730	G	A	10	10	1.6E-12	A	10	8	1.5E-08		Mutation	1.5E-08
662822	G	A	21	12	2.8E-08	A	19	18	4.3E-21		Mutation	2.8E-08
731542	C	T	13	9	2.9E-08	T	13	11	4.1E-12		Mutation	2.9E-08
412934	G	A	8	7	3.9E-08	A	10	8	1.5E-08		Mutation	5.3E-08
2104411	C	T	11	8	8.2E-08	T	12	10	6.1E-11		Mutation	8.2E-08
4189314	G	A	15	13	1.8E-14	A	14	9	1.5E-07		Mutation	1.5E-07
3364045	C	T	14	9	1.5E-07	T	15	12	1.6E-12		Mutation	1.5E-07
2795479	C	T	6	6	1.0E-07	T	6	6	1.0E-07		Mutation	2.1E-07
555449	G	A	9	7	2.3E-07	A	9	9	2.5E-11		Mutation	2.3E-07
1306236	G	A	12	8	4.5E-07	A	12	10	6.1E-11		Mutation	4.5E-07
4173104	G	A	9	7	2.3E-07	A	9	7	2.3E-07		Mutation	4.6E-07
2260312	C	T	8	7	3.9E-08	T	7	6	6.3E-07		Mutation	6.7E-07
2873628	G	A	6	6	1.0E-07	A	7	6	6.3E-07		Mutation	7.4E-07
1219556	C	T	9	7	2.3E-07	T	7	6	6.3E-07		Mutation	8.6E-07
3929806	C	T	17	13	6.1E-13	T	18	10	1.2E-06		Mutation	1.2E-06
719703	G	A	10	7	1.3E-06	A	9	8	2.4E-09		Mutation	1.3E-06
767167	C	T	10	7	1.3E-06	T	11	8	8.2E-08		Mutation	1.4E-06
4671425	C	T	5	5	1.7E-06	T	7	7	6.3E-09		Mutation	1.7E-06
74626	G	A	5	5	1.7E-06	A	6	6	1.0E-07		Mutation	1.8E-06
1556611	G	A	5	5	1.7E-06	A	6	6	1.0E-07		Mutation	1.8E-06
3771665	G	A	13	8	2.3E-06	A	13	10	3.5E-10		Mutation	2.3E-06
1277370	C	T	10	7	1.3E-06	T	10	7	1.3E-06		Mutation	2.6E-06
2831234	G	A	8	6	3.7E-06	A	8	8	4.0E-10		Mutation	3.7E-06
4834248	G	A	16	9	3.8E-06	A	17	15	8.4E-17		Mutation	3.8E-06
4640576	G	A	12	10	6.1E-11	A	11	7	6.9E-06		Mutation	6.9E-06
314407	C	T	11	7	6.9E-06	T	10	9	1.6E-10		Mutation	6.9E-06
1799318	G	A	9	8	2.4E-09	A	6	5	1.1E-05		Mutation	1.1E-05
2647267	T	C	6	5	1.1E-05	C	7	7	6.3E-09		Mutation	1.1E-05
1579929	G	A	6	6	1.0E-07	A	6	5	1.1E-05		Mutation	1.1E-05
2458998	C	T	6	6	1.0E-07	T	6	5	1.1E-05		Mutation	1.1E-05
3936247	C	T	14	13	3.0E-15	T	14	8	1.2E-05		Mutation	1.2E-05
4121383	G	A	14	8	1.2E-05	A	14	9	1.5E-07		Mutation	1.2E-05
1511517	G	A	5	5	1.7E-06	A	6	5	1.1E-05		Mutation	1.2E-05
2963125	G	A	5	5	1.7E-06	A	6	5	1.1E-05		Mutation	1.2E-05
2953567	G	A	9	8	2.4E-09	A	9	6	2.1E-05		Mutation	2.1E-05
4521210	G	A	9	6	2.1E-05	A	10	7	1.3E-06		Mutation	2.2E-05
1066165	C	T	4	4	3.0E-05	T	5	5	1.7E-06		Mutation	3.2E-05
4377924	G	A	5	5	1.7E-06	A	4	4	3.0E-05		Mutation	3.2E-05
655040	C	T	12	7	3.6E-05	T	12	11	6.7E-13		Mutation	3.6E-05
3801057	G	A	12	7	3.6E-05	A	12	8	4.5E-07		Mutation	3.6E-05
1064555	G	A	4	4	3.0E-05	A	6	5	1.1E-05		Mutation	4.1E-05
3156134	C	T	15	8	5.6E-05	T	12	8	4.5E-07		Mutation	5.7E-05
1090650	C	T	7	5	6.1E-05	T	8	8	4.0E-10		Mutation	6.1E-05
4836541	T	C	7	5	6.1E-05	C	8	8	4.0E-10		Mutation	6.1E-05
3417592	G	A	7	5	6.1E-05	A	8	7	3.9E-08		Mutation	6.1E-05
3188210	G	A	6	6	1.0E-07	A	7	5	6.1E-05		Mutation	6.1E-05
700494	C	T	7	5	6.1E-05	T	7	6	6.3E-07		Mutation	6.2E-05
3298937	C	T	7	6	6.3E-07	T	7	5	6.1E-05		Mutation	6.2E-05
496768	A	G	7	5	6.1E-05	G	5	5	1.7E-06		Mutation	6.3E-05
630974	G	A	7	5	6.1E-05	A	7	5	6.1E-05		Mutation	1.2E-04
4169252	G	A	5	4	1.8E-04	A	6	6	1.0E-07		Mutation	1.8E-04
2123568	G	A	5	4	1.8E-04	A	6	5	1.1E-05		Mutation	1.9E-04
3795698	G	A	6	5	1.1E-05	A	5	4	1.8E-04		Mutation	1.9E-04
1779923	G	A	7	5	6.1E-05	A	5	4	1.8E-04		Mutation	2.4E-04
3668382	G	A	19	9	3.1E-04	A	20	16	2.0E-16		Mutation	3.1E-04
2989782	G	A	8	5	3.4E-04	A	8	8	4.0E-10		Mutation	3.4E-04
1297655	C	T	8	5	3.4E-04	T	8	7	3.9E-08		Mutation	3.4E-04
3625847	A	G	8	5	3.4E-04	G	10	7	1.3E-06		Mutation	3.4E-04
4660505	G	A	17	8	9.7E-04	A	20	13	8.6E-11		Mutation	9.7E-04
4586383	C	T	6	4	1.0E-03	T	6	6	1.0E-07		Mutation	1.0E-03
2325510	C	T	5	4	1.8E-04	T	4	3	3.2E-03		Mutation	3.4E-03
4111137	G	A	5	4	1.8E-04	A	4	3	3.2E-03		Mutation	3.4E-03
2901163	C	T	8	5	3.4E-04	T	7	4	5.7E-03		Mutation	6.0E-03
3551802	G	A	7	4	5.7E-03	A	8	5	3.4E-04		Mutation	6.0E-03
4469079	C	T	10	8	1.5E-08	T	10	5	9.1E-03		Mutation	9.1E-03
4539546	G	A	5	3	0.02	A	6	4	1.0E-03		Mutation	0.02
4539738	G	A	5	5	1.7E-06	A	3	2	0.06		Mutation	0.06
2955452	G	A	6	3	0.10	A	7	7	6.3E-09		Mutation	0.10
4153066	G	A	8	6	3.7E-06	A	6	3	0.10		Mutation	0.10
4767697	C	T	6	5	1.1E-05	T	6	3	0.10		Mutation	0.10
4128014	C	T	6	3	0.10	T	6	4	1.0E-03		Mutation	0.10
2410269	G	A	7	3	0.23	A	8	5	3.4E-04		Mutation	0.23
3010834	C	C	18	16	5.8E-18	T	15	15	2.1E-18		Mismatch	0
3615885	C	C	21	21	2.1E-25	T	21	17	1.4E-17		Mismatch	0
4623405	A	T	71	63	3.4E-67	A	71	66	3.6E-73		Mismatch	0
2499952	G	A	15	14	2.0E-16	G	15	15	2.1E-18		Mismatch	2.2E-16
4452587	G	A	10	10	1.6E-12	G	10	9	1.6E-10		Mismatch	1.6E-10
3911612	C	C	21	13	4.2E-10	T	20	15	1.6E-14		Mismatch	4.2E-10
2608981	C	C	7	6	6.3E-07	T	8	8	4.0E-10		Mismatch	6.3E-07
128045	G	C	14	12	2.7E-13	G	12	7	3.6E-05		Mismatch	3.6E-05
3890711	G	A	7	4	5.7E-03	G	7	4	5.7E-03		Mismatch	0.01
2691271	C	T	7	7	6.3E-09	C or -	8	2			Damage	
2750772	G	-	4	3	3.2E-03	A	5	5	1.7E-06		Damage	
3648312	A	A or -	6	2		G	5	4	1.8E-04		Damage	
4329658	C	T	5	4	1.8E-04	T,G,C,-	4	1			Damage	
1412330	G									original allele	No	
2298627	A									original allele	No	
2846790	G									original allele	No	
3386508	G									original allele	No	
3386511	T									original allele	No	
171648	C									No mutation	No	
357352	A									No mutation	No	
992221	G									No mutation	No	
4831490	C									No mutation	No	
1291152	G									edge of map	No	
3591684	C									edge of map	No	
4010951	G									edge of map	No	
4065759	G									edge of map	No	
4314300	G									edge of map	No	

^*^Probability that the real allele is not the most dominant allele

^**^Probability that the Judgement is not correct

**Table 4:** No. of base substitutions after checking original fastq files

	Control	ENU
Real mutation	0	132
Mismatch	7	9
Possibly DNA damage	0	4
No mutation	12	15
Total	19	160

**Fig. 3:**
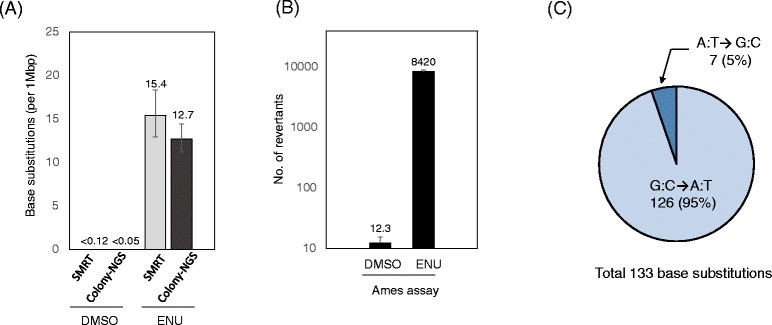
The SMRT method successfully detects ENU-induced base substitution mutations, with a very low background level. **a** Frequency of base substitutions estimated by the SMRT method and the Colony-NGS method. **b** Results of the Ames assay. **c** Mutation spectrum of ENU-induced base substitutions estimated by the SMRT method

We compared the mutation data by this method (SMRT method) with our previous result from colony isolation and whole-genome sequencing (Colony-NGS method). In the ENU-treated samples, the mutation frequencies estimated by the SMRT method (15.4/Mbp) and the Colony-NGS method (12.7/Mbp) were very similar and not significantly different by the binomial test (Fig. 3a). The mutation spectrum obtained by the SMRT method showed that 95% were G:C → A:T transitions and 5% were A:T → G:C transitions (Table 3 and Fig. 3c). This mutation spectrum is well consistent with the ENU signature shown in a previous report [10] and our previous data obtained by the Colony-NGS method (unpublished observations). As for the control (DMSO treated) samples, no mutation was observed in both the SMRT and Colony-NGS methods, thus the mutation frequency was calculated as less than 0.12 per Mbp (1 mutation/8.09 Mbp) and less than 0.05 per Mbp (1 mutation/19.6 Mbp), respectively (Fig. 3a).

## Discussions

In this paper, we successfully detected ultra-low frequency base substitution mutations by using a single-molecule real-time sequencer with the SMRTbell strategy. In principle, this strategy is applicable to any DNA samples such as from bacteria, cell lines, tissues of experimental animals, specimens from patients, and enables us to quantify the mutation frequency and the mutation signature of such DNA samples.

The significant merit for using SMRTbell strategy is that we can sequence each plus and minus strand of a double stranded DNA, thus we are able to distinguish ‘real mutations’ from ‘mismatches’ or ‘DNA damages’. Intriguingly, we could detect not only fixed mutations but also mismatches in the *Salmonella* DNA. In this current procedure, a half of the total mismatches are expected to be detected. From our data, the occurrence of the mismatches in the *Salmonella* genome was roughly estimated as 8 - 10. However, to quantify mismatches absolutely, a new bioinformatics tool should be developed. We also detected 4 possible ‘DNA damages’ only in the ENU-treated sample (Table 4). In Table 3, the raw read judged as ‘Damage’ seems to have lower coverage number than ‘mutation’ or ‘mismatch’. This would reflect the presence of the DNA damages in the SMRTbell templates. Note that, the current procedure is not designed for detection of the DNA damages, thus the detected number would be far less than that of real DNA damages.

The background mutation frequency of the SMRT method in this study was less than 0.12 per Mbp which was comparable to the background level of ‘Duplex Sequencing’ methodologies [2,3]. The background level would depend on the threshold of pass time and accuracy of the CCS. The threshold values used in this study were the most strict values in the current version of PacBio’s instrument control and SMRT Analysis software. The real mutation frequency of the control sample was estimated by combining the Colony-NGS and Ames assay results. In the Ames assay using the same exposure procedure, the mutation frequency of the control sample was 1/685 of that of the ENU-treated sample (Fig. 3b), thus the mutation frequency of the control sample was estimated as 12.7/685 = 0.02 per Mbp. Therefore, more sequencing data (at least 50 Mbp) are required to detect mutations in the control sample.

As for insertion and deletion type mutations, this strategy cannot be used at present because of the very high background level of indels. The reason why more deletions were observed in the ENU-treated sample may be because remaining DNA damages influenced the sequence reaction. Ongoing improvements to the hardware and software of the SMRT sequencer and to the bioinformatics of mutation detection will likely overcome this problem in the near future.

## Conclusion

Ultra-low frequency base-substitution mutations can be detected directly by using the SMRT DNA sequencer, and this technology provides a useful phenotype-independent mutation assay.

## Availability of supporting data

The sequence data used in this study are available at ‘DDBJ Sequence Read Archive’ with the following accounts.

Submission: DRA003525

BioProject: PRJDB3888

BioSample: SAMD00029313 (data of DMSO-treated sample), SAMD00029314 (data of ENU-treated sample).
